# Compression after Impact Behaviour and Failure Analysis of Nanosilica-Toughened Thin Epoxy/GFRP Composite Laminates

**DOI:** 10.3390/ma12193057

**Published:** 2019-09-20

**Authors:** L. Prince Jeya Lal, S. Ramesh, S. Parasuraman, Elango Natarajan, I. Elamvazuthi

**Affiliations:** 1 Department of Mechanical Engineering, KCG College of Technology, Chennai 600 097, India; prince.mech@kcgcollege.com; 2 Department of Mechanical Engineering, School of Engineering, Presidency University, Bangalore 560 064, India; ramesh_1968in@yahoo.com; 3 School of Engineering, Monash University Malaysia, Jalan Lagoon Selatan, Bandar Sunway 46150, Selangor, Malaysia; 4 Faculty of Engineering, UCSI University, Kuala Lumpur 56000, Malaysia; cad.elango.n@gmail.com; 5 Smart Assistive and Rehabilitative Technology (SMART) Research Group, Department of Electrical and Electronic Engineering, Universiti Teknologi Petronas, Bandar Seri Iskandar 32610, Malaysia

**Keywords:** GFRP composites, secondary reinforcement, nanosilica fillers, CAI behaviour, damage assessment

## Abstract

Nanosilica particles were utilized as secondary reinforcement to enhance the strength of the epoxy resin matrix. Thin glass fibre reinforced polymer (GFRP) composite laminates of 3 ± 0.25 mm were developed with E-Glass mats of 610 GSM and LY556 epoxy resin. Nanosilica fillers were mixed with epoxy resin in the order of 0.25, 0.5, 0.75 and 1 wt% through mechanical stirring followed by an ultrasonication method. Thereafter, the damage was induced on toughened laminates through low-velocity drop weight impact tests and the induced damage was assessed through an image analysis tool. The residual compression strength of the impacted laminates was assessed through compression after impact (CAI) experiments. Laminates with nanosilica as secondary reinforcement exhibited enhanced compression strength, stiffness, and damage suppression. Results of Fourier-transform infrared spectroscopy revealed that physical toughening mechanisms enhanced the strength of the nanoparticle-reinforced composite. Failure analysis of the damaged area through scanning electron microscopy (SEM) evidenced the presence of key toughening mechanisms like damage containment through micro-cracks, enhanced fiber-matrix bonding, and load transfer.

## 1. Introduction

Glass fiber reinforced polymer (GFRP) composite materials are extensively used in aerospace, automobile, wind turbine, and marine applications due to its high stiffness, lightweight, excellent corrosion, and fatigue resistance properties. However, GFRP composites are vulnerable to accidental and in-service damages leading to barely visible impact damages caused due to impact events [1,2,3]. Cracking of the matrix system, delamination at the fiber-matrix interface, fiber pull-out, and fracture are a few frequently observed barely visible failure modes in GFRP materials leading to the reduction of strength and stiffness of the structure. During an impact event, the predominant mode of failure is the matrix cracking which occurs within the plies due to the shear stresses induced through the thickness. Subsequently, a coalescence of these micro-cracks in the matrix system serves as the source for delamination within the plies which is due to the mode II interlaminar shear stresses [4]. Therefore, it is essential to enhance the strength of the matrix system through a sustainable technique to prevent matrix cracking in composite structures.

An advanced technique like impregnating self-healing microcapsules was previously attempted and it was reported that the tensile and flexural strength of composites were dependent on the size of microcapsules. In continuity to the earlier work reported, the machinability of the self-healing GFRP composite laminates was carried out to understand the size and concentration effects of the healing agent [5,6]. A novel technique to enhance the impact resistance of the composite laminates is through reinforcing micro and nano-sized fillers into the matrix system. Significant enhancement in fracture toughness of the matrix system was observed in various cases through key toughening mechanisms like crack pinning, crack deflection, crack bridging, and filler debonding [7,8]. Researchers endeavored to use nanofillers, like single-walled carbon nanotubes (SWCNT), double- walled carbon nanotubes (DWCNT), multi-walled carbon nanotubes (MWCNT) [9,10,11,12], graphene [13,14,15], nanoclay [16,17,18], and nanosilica [19,20,21] into various polymers and reported the effect of reinforcement in polymer composites. However, reinforcing nanofillers into the polymer matrix does not always enhance the fracture toughness and modulus of the composite material. A few researchers have encountered setbacks in accomplishing a homogeneous dispersion of nanofillers into the base matrix. The nanofillers agglomeration does not help to increase any mechanical property of the composite. To achieve a homogeneous dispersion of nanofillers in the matrix, it is inevitable to use an expensive surface functionalization process [22,23,24]. Dispersion behavior of nanofillers in the matrix can be investigated through various techniques like Fourier-transform infrared spectroscopy (FTIR), scanning electron microscopy (SEM), transmission electron microscopy (TEM), and atomic force microscopy (AFM) [25,26].

Selected research works were done on the tensile, flexural, and impact behavior of nanosilica- reinforced composite materials. Gong et al. [19] synthesized nanosilica fillers of 80 nm by a sol-gel technique and achieved a homogeneous dispersion through mechanical blending and enhanced interfacial bonding between glass fiber and modified epoxy. Fathy et al. [20] synthesized silica nanoparticles of a size in the order of 10–20 nm and examined the fatigue performance of nanosilica-reinforced glass fiber composite laminates. Johnsen et al. [21] investigated the influence of nanofillers of different sizes as toughening agents and also explored the associated toughening mechanisms. Krushnamurthy et al. [18] investigated the effect of nanoclay on the toughness behavior of E-glass composite laminates. It was noted that the toughness of the epoxy composite laminates increased by 25% at 3 wt% of nanoclay. Likewise, flexural strength and tensile strength of the E-glass/epoxy composite laminates were enhanced by 12% and 11%, respectively. They also observed a decrease in properties at 5 wt% due to matrix embrittlement. Interestingly, it was observed that matrix embrittlement is beneficial to increase the impact mitigation by 29%. Kostopoulos et al. [27] analyzed the effect of reinforcing polycaprolactone nanofibers in epoxy matrix by performing impact experiments on glass fiber/epoxy composites. A numerical model was developed and validated with experimental results. The results of the study revealed that composite laminates reinforced with nanofibers exhibited enhanced resistance to impact damage. Garcia et al. [28] used nylon nanofibers to enhance the performance of the matrix system and the delamination resistance and interlaminar strength of glass fiber-reinforced polymer composites with and without nylon nanofibers were investigated.

The post-impact residual compression strength of the composite structure is one of the design variables that need to be evaluated to ensure the structural integrity of the composite structure. Caminero et al. [29] worked on the influence of thickness and ply-stacking sequence on the compression after impact strength of carbon fiber-reinforced epoxy laminates and observed that angle-ply laminates depict better performance in terms of damage tolerance. A similar study was carried out by [30] to assess the damage behavior of 3D printed composite laminates through a phased array ultrasonic technique. Literature regarding post-impact compression behavior of nanofiller-reinforced composite laminates was not available and hence an attempt was made to study the effect of reinforcing nanofillers on the residual strength of GFRP laminates post-impact. Low-velocity drop weight impact test methods were selected to induce barely visible surface damage and subsurface invisible damage. Induced surface damages were assessed by an image analysis technique and thereafter, the residual compression strength of the nanosilica-toughened laminates were assessed through compression after impact tests. The investigated results were compared with the pristine composites.

## 2. Materials and Methods

### 2.1. Materials and Fabrication of Composite Laminates

Thin GFRP composite laminates of 3 ± 0.25 mm were fabricated from plain woven roving E-Glass mats of 610 GSM and LY556 Epoxy resin with HY951 hardener. Nanosilica fillers of 17 nm exhibiting lower matrix embrittlement were utilized as secondary reinforcement. Initially, nanosilica fillers were mixed into the epoxy resin in the order of 0.25, 0.5, 0.75, and 1 wt% through mechanical stirring, followed by an ultrasonication method. The melt viscosity of the matrix increased with the increase in nanosilica wt% and a higher wt% (above 1 wt%) lead to agglomerations of nanoparticles. Up to 1% of nanosilica loading, the formation of agglomerations were controlled by preheating the matrix and nanoparticles. The reinforced matrix was subsequently degassed to remove air bubbles that were generated during the blending process [31]. Thereafter, to initiate the curing process, hardener was added to the modified matrix in the ratio of 1:10 by weight. Laminates were developed by a compression molding technique. ASTM D7137M-12 compression after impact rectangular specimens of size 150 mm × 100 mm were cut from the fabricated plates using an abrasive water jet cutting machine. Cutting parameters were set as suggested in [32] to avoid any structural damage to the sample edges.

### 2.2. Fourier-Transform Infrared (FTIR) Spectroscopy 

FTIR spectroscopy is an analytical procedure to recognize organic, polymeric, and in some cases, inorganic materials. A Perkin Elmer Spectrum FTIR instrument (Waltham, MA, USA) was utilized to capture the spectrums of pristine and nanosilica-reinforced polymer composites. The instrument consists of a globar and mercury vapor lamp as sources, an interferometer chamber comprising of KBr and mylar beam splitters followed by a sample chamber and detector. It is able to cover the entire region of 450–4000 cm^−1^ with a typical resolution of 1 cm^−1^. The spectrometer was operated under purged conditions.

### 2.3. Low-Velocity Drop Weight Impact Tests

**Figure 1:**
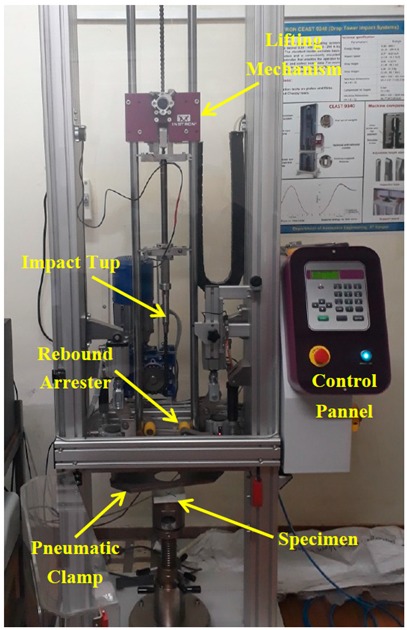
Instrumented low-velocity impact test facility.

An instrumented Ceast Fractovis 9340 (Instron, Norwood, MA, USA) drop weight impact tester fitted with a rebound arrester as shown in Figure 1 was utilized to assess the low-velocity impact response of composite laminates. The specimens were impacted exactly at the center with a hemispherical impactor tup of 16 mm diameter. Figure S1a presents the test parameters of low-velocity impact experiments to achieve the impact energy of 10 J. The total mass of the impactor was kept constant for all the experiments. Specimens were held rigidly in the fixture using a pneumatically-operated clamp. The clamping system has an outer diameter of 110 mm, inner diameter of 70 mm, and a clamping force of 1000 N. Parameters such as impact force, impact energy, impact velocity, and deformation were measured using a 45 kN load cell during the conduct of impact tests and were recorded using a CEAST DAS 64K data acquisition system (Instron, Norwood, MA, USA) with the sampling frequency rate of 4 MHz interfaced with Visual IMPACT Version 6. Data Acquisition System parameters are displayed in Figure S1b.

### 2.4. Damage Assessment by Image Analysis

**Figure 2:**
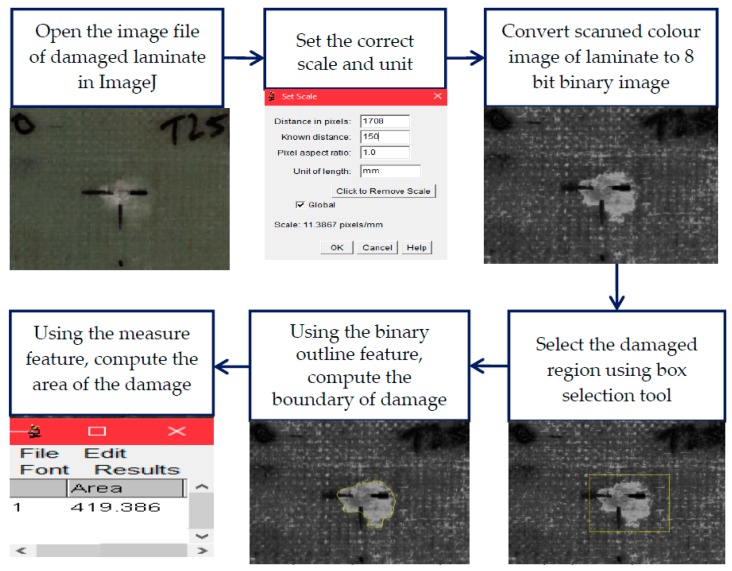
Image analysis technique used to compute the damaged area.

The damaged area that developed on the laminates due to low-velocity impact was analyzed through ImageJ, an image analysis software (LOCI, University of Wisconsin). The image analysis technique used to compute the damaged area of composite laminates is shown below in Figure 2.

### 2.5. Compression after Impact (CAI) Tests

The residual compression strength of composite laminates post-impact was assessed through CAI tests at room temperature as per ASTM D7137 M-12 standard using a Universal Testing Machine with 100 kN capacity (Tinius Olsen, Horsham, PA, USA). The CAI specimens were clamped exactly on the fixture by adjusting four supporting plates for arresting the global buckling. The compressive load was applied under a constant displacement rate of 0.5 mm/min since the mechanical response of FRP composites depends strongly on the displacement rate [33,34]. The data acquisition system in the universal testing machine recorded the force-displacement of the experiments.

### 2.6. Failure Analysis Using SEM

Interfacial bonding analysis and fracture analysis were carried out using a Carl Zeiss SUPRA-55 Variable Pressure Field Emission Scanning Electron Microscope (ZEISS, Oberkochen, Germany) operated at extremely high voltages (from 0.2 kV) and capable of producing high magnification images. Failure mechanisms like fiber fracture, fiber pull-out, matrix cracking, delamination, and micro-cracks were detected.

## 3. Results

### 3.1. FTIR Spectroscopy Analysis

**Figure 3:**
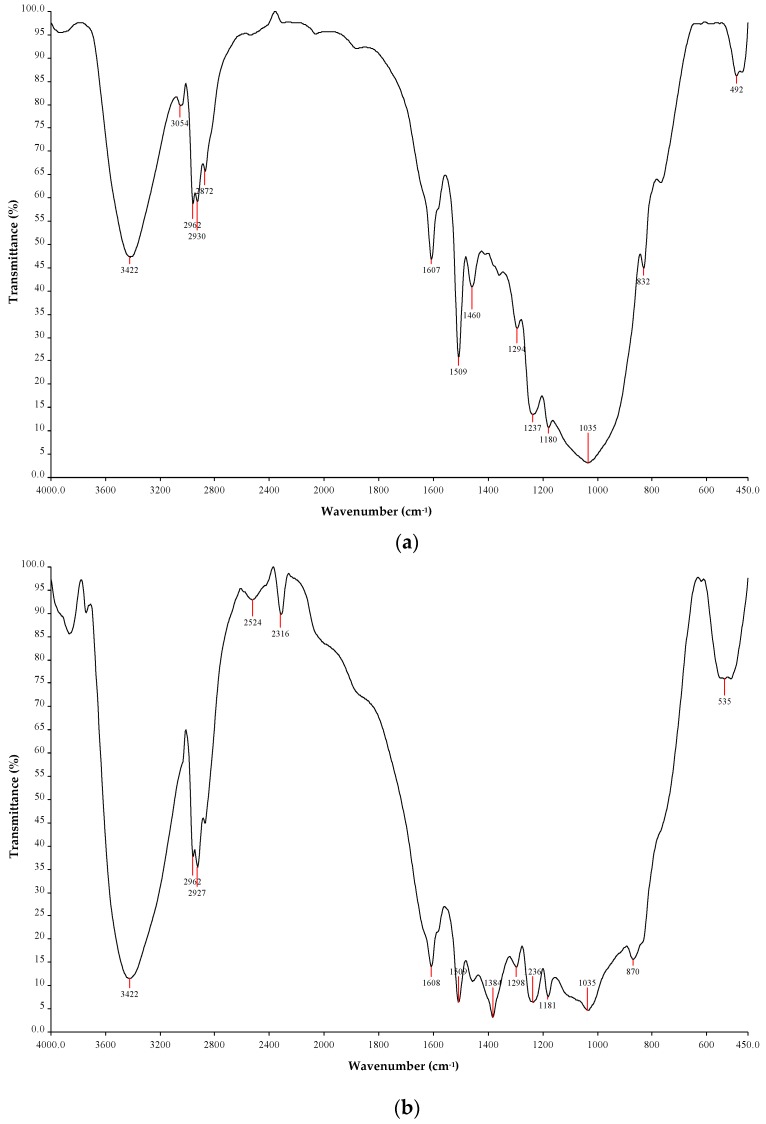
(**a**) FTIR spectrum of pristine epoxy resin; (**b**) FTIR spectrum of 0.75 wt% nanosilica-loaded epoxy resin.

The FTIR spectrum of pristine epoxy is shown in Figure 3a. The FTIR spectrum of pristine epoxy resin displayed many peaks in the mid-IR region pertaining to various types of stretching and bending vibrations of the bonds. The strong band at 3422 cm^−1^ was due to O–H stretching vibrations of the epoxy group. The band at 3054 cm^−1^ corresponded to C–H tension of the methyl group generally observed in low polymerized resins. The peaks at 2962 to 2872 cm^−1^ attributed to C–H fundamental stretching vibrations. The peaks at 1607 and 1509 cm^−1^ corresponded to stretching of C=C and the C–C aromatic rings. The bands between 1300 to 1035 cm^−1^ corresponded to stretching vibrations of the C–O–C rings of epoxy resin. The peak at 832 cm^−1^ is due to the stretching of the C–O–C oxirane group. The FTIR spectrum of 0.75 wt% silica-loaded epoxy resin is shown in Figure 3b. It can be observed from the spectrum that the bands observed in pure epoxy resin were retained in the spectra of nanosilica-loaded epoxy resin. The absorption peaks of nanosilica will be observed around 950 and 450 cm^−1^. However, in our study, the epoxy resin has strong bands at 950 and 450 cm^−1^ which overshadowed the nanosilica peaks. These observations revealed that the dispersion of nanosilica in epoxy resin did not change the characteristics of epoxy and hence the properties were retained. It also suggests that there was no chemical reaction between the nanosilica and epoxy and the bonding between the base matrix and nanofillers was due to a physical process. 

### 3.2. Responses of Low-Velocity Drop Weight Impact Tests

**Figure 4:**
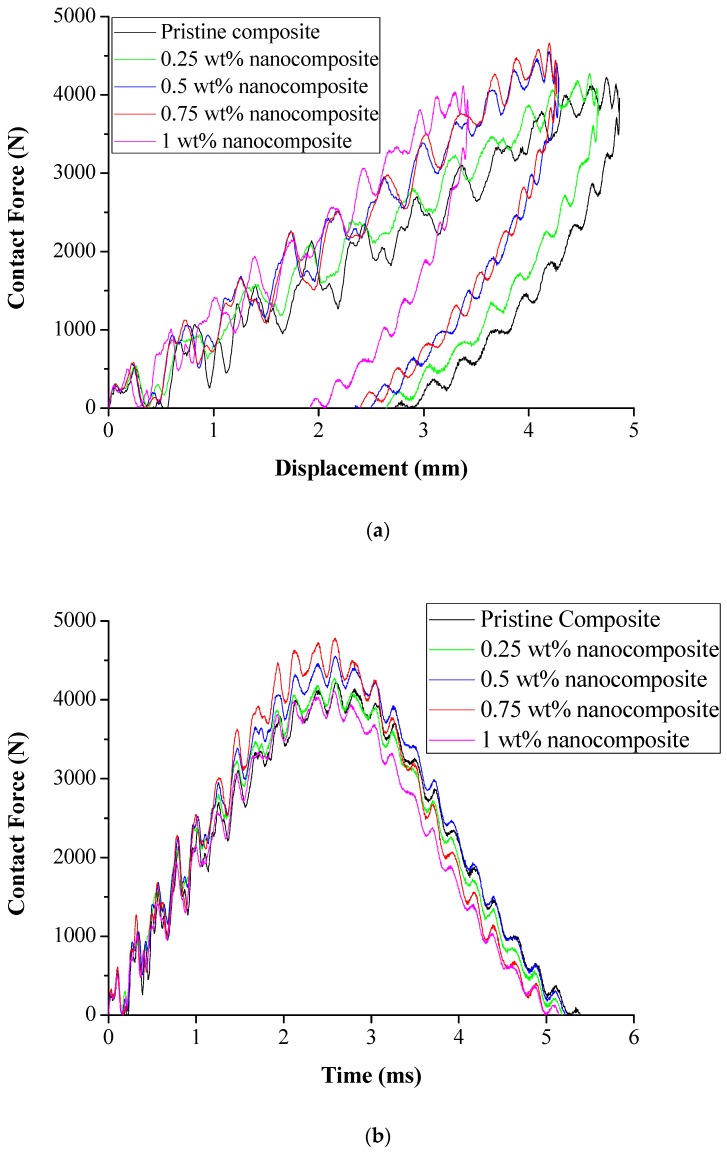
Responses of low-velocity impact (**a**) Contact force versus displacement behavior of composite laminates. (**b**) Contact force versus time history of composite laminates.

The responses of low-velocity impact experiments were acquired through an instrumented drop weight impact system for pristine and nanosilica-toughed composites. Figure 4 presents the results of the instrumented drop weight impact tests.

Figure 4a shows the rebound of the impactor after the impact event was witnessed which emphasizes no through-penetration of tup in laminates [35]. Figure 4b shows that it is observed that during a low-velocity impact, contact force-time response is symmetric during impact and rebound. A steady rise in contact forces is observed until it reaches the peak contact force. A drop in contact forces is thereafter witnessed for all the specimens. Pristine composites registered a peak contact force of 4226 N and toughened composites recorded a peak contact force in the order of 4275 N, 4552 N, 4582 N, and 4116 N for 0.25 wt%, 0.5 wt%, 0.75 wt%, and 1 wt%, respectively. Also it was noted that the duration of impact was reduced with the increase in nanosilica content. Energy absorbed by the laminates was determined from the force-displacement curve generated during the impact event. Energy transferred to the test laminates were in the order of 7.11 J for pristine composites and 7.13 J, 7.66 J, 7.74 J, and 6.53 J for nanosilica-toughened composite laminates. Energy absorption behavior of toughened composites was in the order of 0.28%, 7.7%, and 8.8% for 0.25 wt%, 0.5 wt%, and 0.75 wt%, respectively. Highly loaded 1 wt% nanofiller-reinforced composite laminates recorded an 8.1% drop in energy absorption compared to pristine composites. Since the properties of the primary reinforcement were constant, it is evident that the enhancement in energy absorption was due to the key toughening mechanisms achieved through the reinforcement of secondary reinforcement.

### 3.3. Assessment of Induced Damage Due to Low-Velocity Impact Experiments

Damage induced on the composite laminates is discussed in this section. The induced damage was computed through an ImageJ processing tool and is compared with the actual image. Thin composite laminates disclose poor resistance to low-velocity impact due to the early failures like transverse matrix cracking and fiber-matrix interface debonding. This drawback can be targeted by strengthening the brittle matrix system by using nanofillers as secondary reinforcements to enhance the interlaminar properties of the brittle epoxy matrix. 

**Figure 5:**
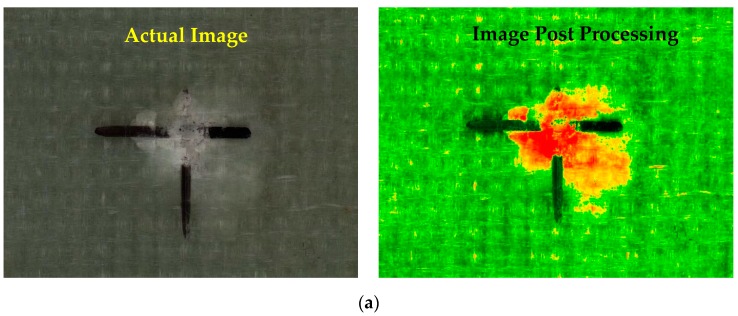

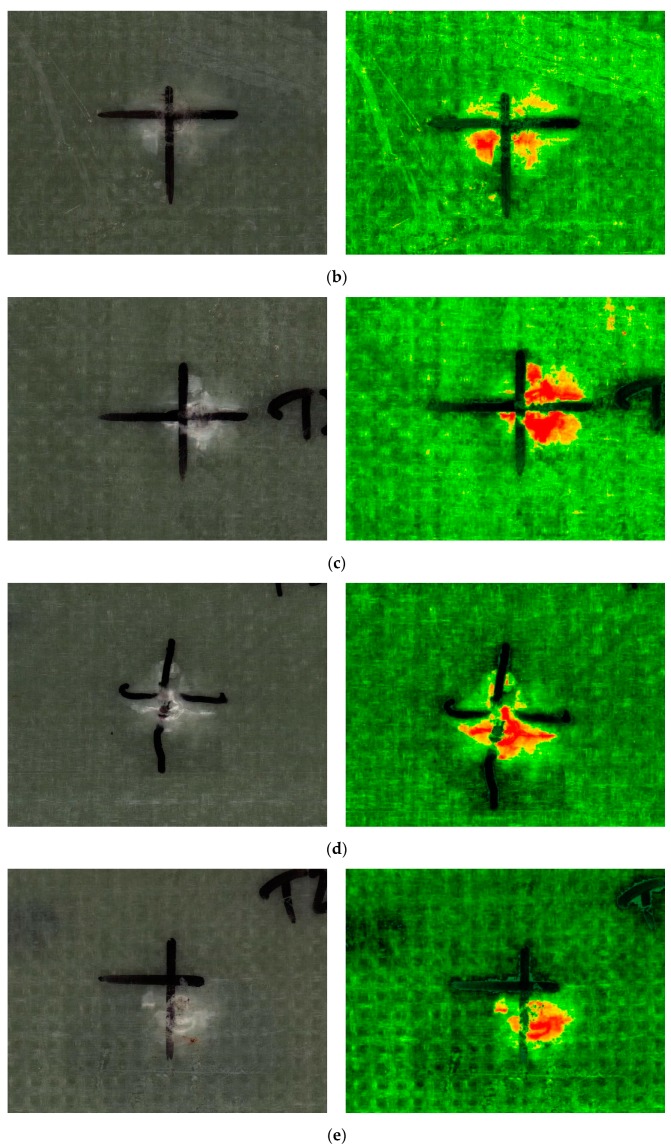
Induced damage due to low-velocity impact in (**a**) Pristine composite; (**b**) 0.25 wt% nanosilica- reinforced composite; (**c**) 0.5 wt% nanosilica-reinforced composite; (**d**) 0.75 wt% nanosilica-reinforced composite; (**e**) 1 wt% nanosilica-reinforced composite.

From the images representing damaged composites, it is evident that the pristine composite suffered the highest damage. Whereas, in toughened composites, nanofillers suppressed the damage propagation in toughened laminates and at the same time, promoted efficient load transfer to primary reinforcements. The permanent damage induced in nanosilica-reinforced composites is witnessed to be less than the pristine composites which highlight the influence of nanofillers in mitigating the damage propagation. From Figure 5a it can be observed that in pristine composites, the damaged area propagated to greater regions. Whereas, in toughened composites, the damage induced was localized and contained to a great extent. The damage induced in pristine composites is computed to be 369 mm^2^, whereas the induced damage area in toughened composites are in the order of 275 mm^2^, 239 mm^2^, 175 mm^2^ and 145 mm^2^ in 0.25 wt%, 0.5 wt%, 0.75 wt%, and 1 wt% nanosilica-toughened composite laminates which are 25.5%, 35.2%, 52.5%, and 60.7% less than pristine composites. From Figure 5a, it is observed that local damage occurred at the impact point which induced the initial matrix failure due to high transverse shear stress. Nanofillers play a vital role in mitigating this initial failure by enhancing the fracture toughness of the matrix system, thus maintaining the structural integrity of the laminate.

### 3.4. Residual Compressive Strength of Low-Velocity Impacted Specimens

Damage induced by low-velocity impact is critical, as often no sign of damage is visible during visual inspection. Hence, the load-bearing competence of the composite specimens after inducing permanent damage through low-velocity impact is studied experimentally through CAI tests and the performance of pristine and nanofiller-toughened composites are compared.

**Figure 6:**
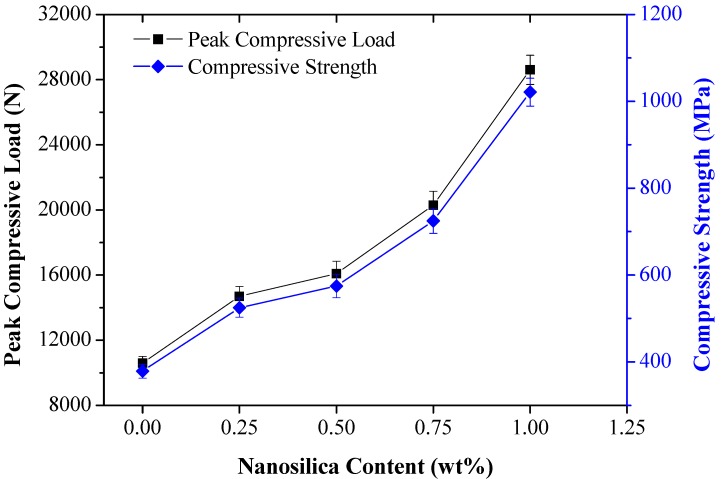
Post-impact compressive behavior of pristine and toughened composites.

Responses of low-velocity impact and CAI properties of pristine and toughened composites are presented in Table 1. The compressive strength of pristine composite laminates post-impact is reduced due to the severe permanent failures induced during impact tests. On the contrary, reduction in induced permanent deformation and enhancement in stiffness was observed in nanosilica-toughened composite laminates. This was due to the constriction in crack propagation from localized damage regions. Under compressive loading, nanofillers bridged the existing micro-cracks, thereby enhancing the load-bearing ability and compression strength. From Figure 6, it is observed that pristine composite laminates offered the lowest compressive strength of 378.57 MPa and a steady rise in compressive strength is recorded for toughened laminates in the order of 525.2 MPa, 575.4 MPa, 725.3 MPa, and 1021.8 MPa with the increase in nanosilica wt%. A significant rise in the compressive strength of toughened composite laminates was observed in the order of 38.81%, 51.99%, 91.5%, and 169.9%.

### 3.5. Fracture Analysis of Laminates by SEM

**Figure 7:**
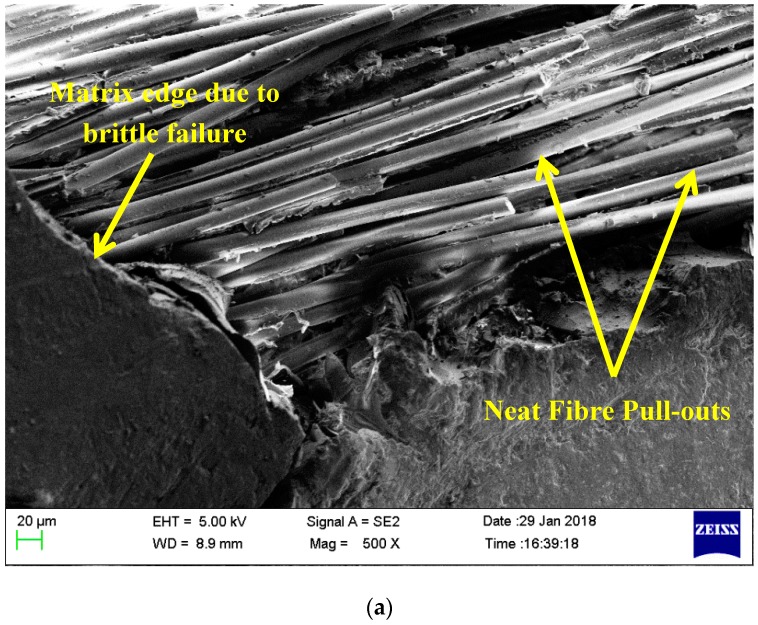

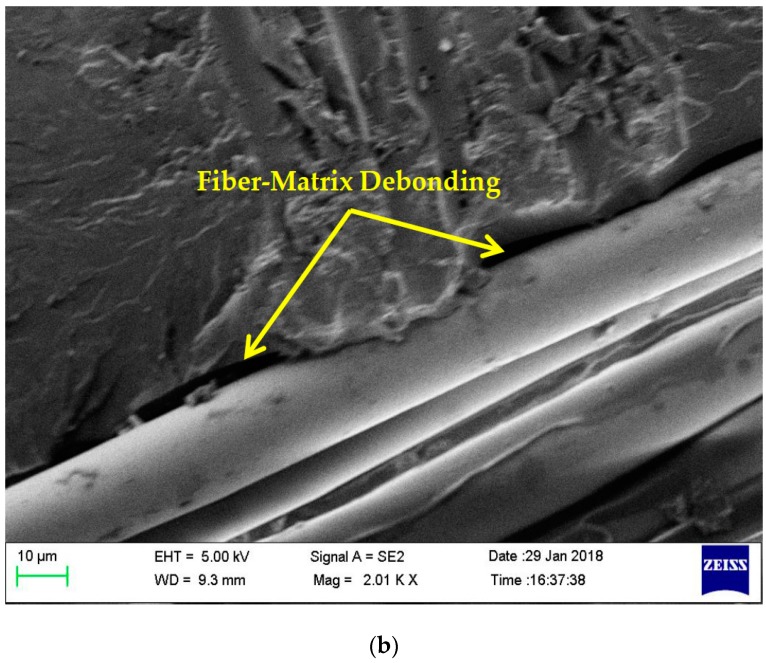
Failure analysis of pristine composites (**a**) Fibre pulled out neatly without fracture and damage (**b**) Poor interfacial bonding between matrix and fibre.

**Figure 8:**
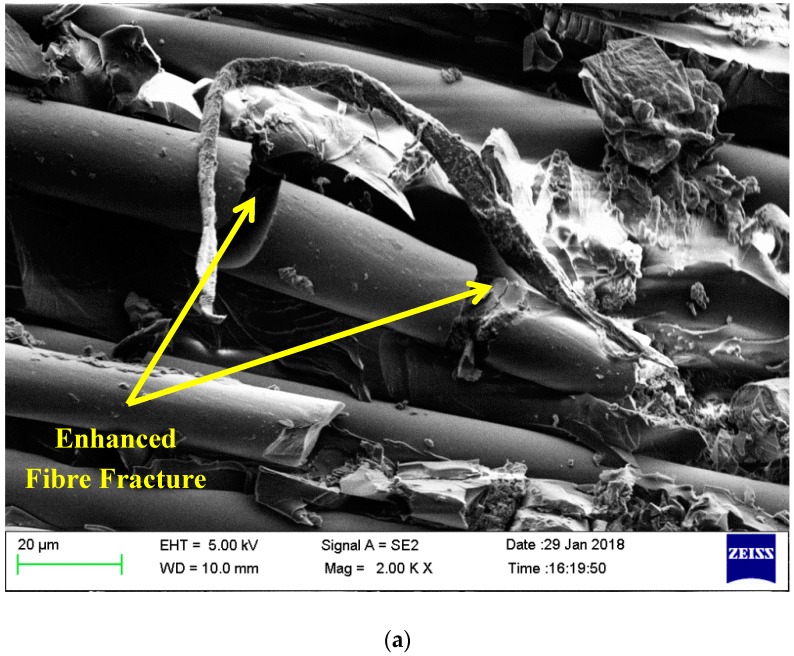

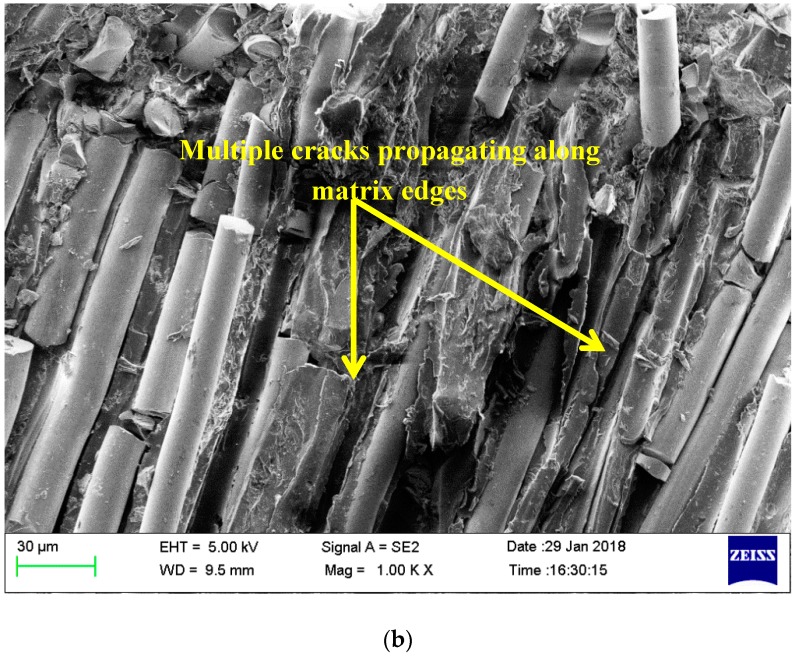
Fracture analysis of toughened composites with 0.75 wt% nanosilica as secondary reinforcements (**a**) Enhanced fibre damage; (**b**) Crack initiation and propagation in matrix.

The interfacial bonding nature and failure behavior of pristine laminates and toughened laminates with 0.75 wt% nanosilica are compared. The brittle failure of the matrix is primarily initiated by the debonding of nanofillers and fibre from the matrix [36]. This is a key toughening mechanism in nanofillers impregnated with epoxy composites. Figure 7 depicts the failure region of pristine composites. Individual fibers of pristine composites are pulled out from the matrix without any fracture. This indicates poor adhesion between the matrix and fiber filaments in neat composites resulting in a weak interfacial bonding. Also, smooth fracture surfaces are evidenced in pristine composites along failed matrix edges. Figure 8 represents the fracture analysis of toughened composites with 0.75 wt% nanosilica as secondary reinforcements highlighting the fracture of fibers witnessing a better load transfer and also highlighting the multiple cracks propagating along the matrix edges. Similar observations are made in [19], which is an effect of utilizing secondary reinforcements.

### 3.6. Key Toughening Mechanisms in Nanosilica-Reinforced GFRP Composite Laminates

Matrix failure is the primary failure mechanism in thermosetting polymer composites. This is due to the brittle behavior of the matrix system. Under loading, micro-matrix cracks develop first. Similar matrix cracks accumulate together to form a localized damage region. Along these failure planes, delamination is induced and propagated due to continuous loading. The load transferred to primary reinforcement through the matrix is uneven due to these localized failures. Hence, a higher amount of load is concentrated in certain regions leading to fiber fracture. This process occurs continuously and finally, all fibers reinforced along the failure direction undergo failure. Nanofillers play a vital role in mitigating this initial failure by enhancing the fracture toughness of the matrix system, thus maintaining the structural integrity of the laminate. The enhancement in the mechanical behavior of nanosilica-reinforced composites could be attributed to the higher strength and toughness of the matrix system loaded with nanosilica. Plastic deformation of the matrix system instigated by micro-cracking, crack propagation, debonding of nanoparticles, fiber pull-out, and fiber fracture are significant toughening mechanisms encountered due to the presence of nanoparticles in the matrix. These toughening mechanisms must consume significant amounts of energy, thus leading to increased stress transfer. 

## 4. Conclusions

The influence of nano-sized silica particles used as secondary reinforcement fillers in glass/epoxy composite laminates was analyzed experimentally through FTIR analysis, low-velocity impact tests, and CAI experiments. Failure surfaces of the damaged laminates were analyzed using SEM studies. The conclusions of this experimental research work are summarized as follows:

- From FTIR spectrums it can be observed that the dispersion of nanosilica particles in epoxy resin did not change the characteristics of epoxy and its properties were retained. It also suggests that there was no chemical reaction between the nanosilica and epoxy and the bonding was just a physical process.
- Responses of low-velocity impact experiments disclosed that nanofiller-toughened composite laminates exhibited enhanced peak load, reduced impact duration, and enhanced energy absorption while suppressing the damage propagation. The use of silica nanofillers enhanced matrix-fibre bonding, enhanced load transfer, and fracture toughness. Toughened composite laminates with 0.75 wt% nanosilica content offered superior results.
- Post-impact compression responses assessed through CAI conveys the effects of nanofillers on the residual compressive strength of the laminates. Significant enhancement in peak load and residual compressive strength was observed in toughened laminates. Composite laminates toughened with 1 wt% silica nanoparticles offered the highest peak load and compressive strength. This was due to the enhanced damage suppression observed during low-velocity impact experiments.
- Failure analysis of damaged surfaces through SEM reported the presence of key toughening mechanisms like the generation of micro-cracks, crack deflection, enhanced fiber-matrix interface, and superior load transfer to primary reinforcements which was frequently noted in nanofiller-toughened polymer materials. This section is not mandatory but can be added to the manuscript if the discussion is unusually long or complex.

## Figures and Tables

**Table 1:** Responses of low-velocity impact.

Responses of Low-Velocity Impact					
Nanosilica Content (wt%)	Peak Contact Force (N)	Absorbed Energy (J)	Damage Area (mm^2^)	Peak Compression Load (N)	Compression Strength (MPa)
0	4226	7.11	369	10,600	378.57
0.25	4275	7.13	275	14,700	525.2
0.5	4552	7.66	239	16,100	575.4
0.75	4582	7.74	175	20,300	725.3
1	4116	6.53	145	28,600	1021.8

## Supplementary Materials

The following are available online at https://www.mdpi.com/1996-1944/12/19/3057/s1, Figure S1: (a) Low velocity impact test parameters (b) Data Acquisition System parameters.
