# Supplementary material for: Compression after Impact Behaviour and Failure Analysis of Nanosilica-Toughened Thin Epoxy/GFRP Composite Laminates
Source: Materials (Basel). 2019 Sep 20;12(19):3057. doi: 10.3390/ma12193057 (PMC6804005; doi:10.3390/ma12193057)
Supplement: Supplementary file 1 [file materials-12-03057-s001.pdf]

materials

Article

Supplementary Materials: Compression after Impact Behaviour and Failure Analysis of Nanosilica Toughened Thin Epoxy/GFRP Composite Laminates

L. Prince Jeya Lal [1], S. Ramesh [2], S. Parasuraman [3,*], Elango Natarajan [4] and I. Elamvazuthi [5,*]

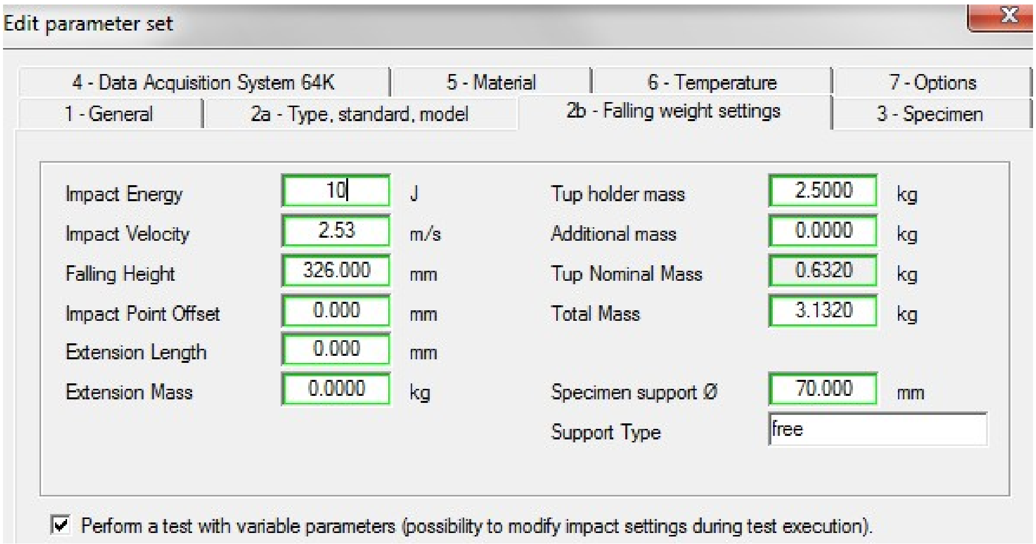

(a)

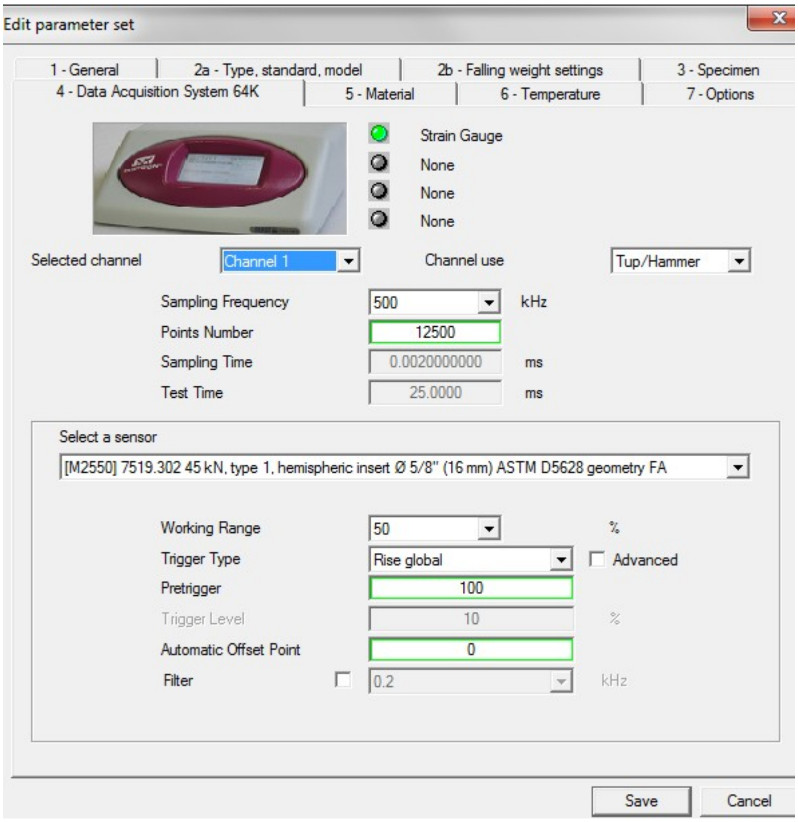

(b)

Figure S1. (**a**) Low velocity impact test parameters (**b**) Data Acquisition System parameters.
